# Coronary artery perforation after bioresorbable scaffold implantation treated with a new generation covered stent—OCT insights

**DOI:** 10.1186/s12872-022-02501-3

**Published:** 2022-02-23

**Authors:** D. Chen, R. Gadeley, A. Wang, N. Jepson

**Affiliations:** 1 Prince of Wales Hospital, Barker Street, Randwick, NSW 2031 Australia; 2 Eastern Heart Clinic, Barker Street, Randwick, NSW 2031 Australia; 3 University of New South Wales, Sydney, NSW 2052 Australia

**Keywords:** Coronary, Artery, Perforation, Bioresorbable, Stent, Optical, Coherence, Tomography, Case report

## Abstract

**Background:**

Coronary artery perforation is a rare but potentially lethal complication of percutaneous coronary intervention (PCI) with an associated mortality of 7–17%. We report the case of coronary artery perforation complicating Absorb bioresorbable vascular scaffold (BVS) implantation and the associated technical challenges with managing this life-threatening complication.

**Case report:**

A 46-year-old male was referred to our institution and underwent PCI with an Absorb bioabsorbable vascular scaffold (BVS) to a proximal LAD long segment bifurcation lesion. Following pre-dilation and deployment of the 3.5 × 28 mm Absorb BVS, high pressure post-dilation of the distal scaffold was complicated by a large, Ellis type III coronary perforation with no flow to the distal LAD beyond the rupture, and associated with a large pericardial effusion confirmed on bedside transthoracic echocardiogram (TTE). The insult was temporised with prolonged balloon inflation within the Absorb BVS immediately proximal to the site of perforation, permitting urgent insertion of a pericardial drain. After deflation of the balloon, a 3.0 × 21 mm BeGraft covered stent was deployed across the perforation, restoring normal LAD flow and abolishing the perforation. Cardio-pulmonary resuscitation was not required and the patient remained conscious throughout the procedure. TTE demonstrated normal left ventricular function and the patient was discharged 3 days later. Repeat angiography at 3 months showed patent stents with TIMI III flow, and optical coherence tomography (OCT) showed good expansion and apposition of the proximal Absorb BVS and BeGraft. The patient has remained well 4 years after PCI with no major cardiovascular events.

**Conclusion:**

The utility of bioresorbable scaffold technology remains controversial although meticulous implantation techniques are associated with improved clinical outcomes. Adoption of the Pre-dilatation, Sizing and Post-dilatation (‘PSP’) method of BVS implantation with routine aggressive vessel preparation and scaffold optimization however may contribute to a higher risk of vessel perforation. The case emphasises the importance of accurate sizing of the vessel with intracoronary imaging and demonstrates the value of newer generation covered stents with single-layer design and slimmer crossing profile producing improved deliverability and procedural success.

**Supplementary Information:**

The online version contains supplementary material available at 10.1186/s12872-022-02501-3.

## Introduction

Coronary artery perforation is a rare but potentially lethal complication of percutaneous coronary intervention, with the overall incidence ranging from 0.19 to 0.59% [1] and an associated mortality of 7–17% [2]. Risk factors for non-guidewire induced coronary perforation in contemporary PCI practice include female sex, increasing age, calcification within the target vessel, treatment of a chronic total occlusion, use of a cutting balloon or rotational atherectomy [2], oversizing of balloons, and high pressure balloon inflations [3]. This article highlights a case of coronary artery perforation following deployment of a bioresorbable vascular scaffold (BVS).

## Case presentation

A 46-year-old male was transferred to our tertiary referral centre for consideration of multi vessel and complex percutaneous coronary intervention (PCI) on a background of hypertension, a 20 pack year history of cigarette smoking, and a significant family history of premature coronary artery disease. Transthoracic echocardiography (TTE) demonstrated normal left ventricular systolic function. Recent coronary angiography 1 week prior had already demonstrated severe disease in the distal right coronary artery (RCA) following a non-ST elevation myocardial infarction (NSTEMI), and the culprit lesion was stented with a Resolute Onyx zotarolimus drug eluting stent (DES) (Medtronic, Minneapolis, USA) at the regional centre from where he had been referred. Persistent stable angina prompted repeat angiography, showing a patent RCA stent with residual disease in the left anterior descending (LAD) and left circumflex (LCX) arteries. Physiological assessment of these arteries however, demonstrated a fractional flow reserve (FFR) ratio of 0.81 in the LCX and 0.64 in the LAD, and the plan was subsequently for PCI to the LAD undertaken in a tertiary referral centre with significant experience using bio-resorbable scaffold technology.

**Fig. 1:**
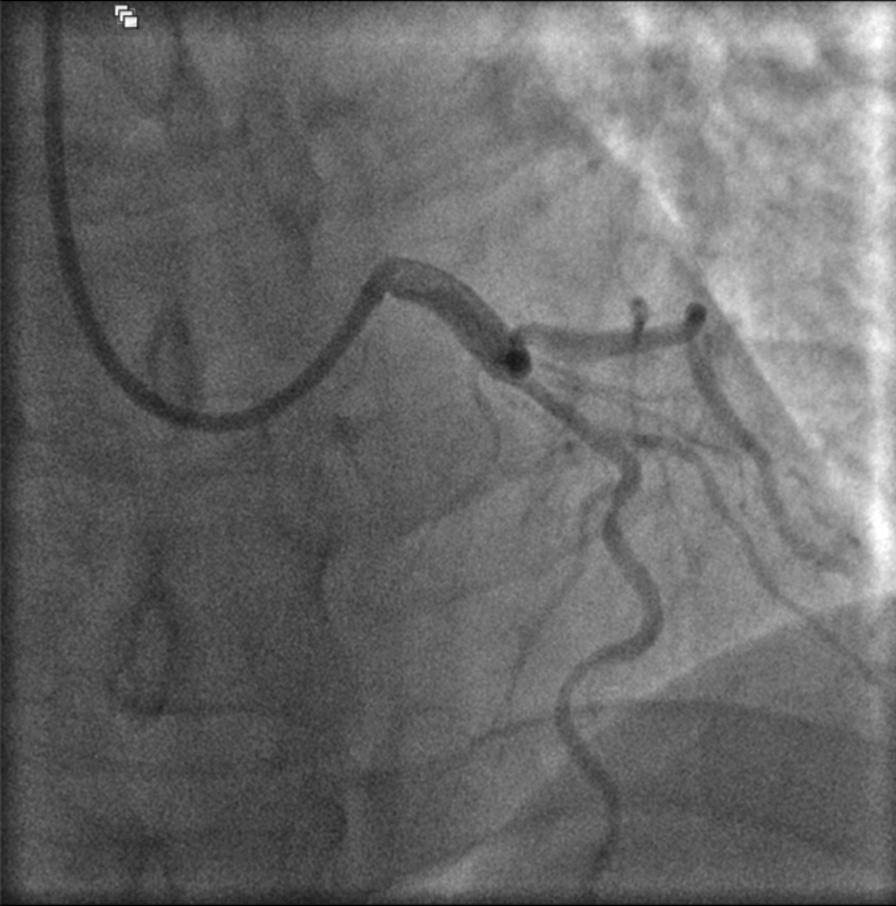
Proximal LAD stenosis involving bifurcation with first diagonal branch (Medina-1, 1, 0) with FFR of 0.64. Given the patient’s young age and suitable target lesion characteristics, it was felt an Absorb BVS was preferred over a permanent metallic implant

The target lesion in the proximal LAD was a long segment and involved the bifurcation with the first diagonal branch (Medina 1, 1, 0) (Fig. 1). In the context of his young age and suitable target lesion characteristics including the lesion length and the mild degree of calcification, an Absorb bioabsorbable vascular scaffold (BVS) (Abbott Vascular, Santa Clara, CA, USA) was therefore selected, avoiding a permanent metallic implant and preserving future revascularisation options. Following right trans-radial access, the LAD and first diagonal branches were wired employing an angiographic-guided, provisional bifurcation PCI strategy using an EBU 4.0 guide catheter (Medtronic, Minneapolis, USA). The mid LAD was pre-dilated with a 3.0 mm Hiryu (Terumo Medical, Tokyo, Japan) non-compliant monorail balloon and given full balloon expansion was achieved, the target lesion was then covered with a 3.5 × 28 mm Absorb BVS implanted at 14 atm (deployed across the diagonal side-branch, protected by a jailed-wire). Although the target lesion appeared longer (than 28 mm), a strategy of proximal-to-distal scaffolding was planned given the proximal landing zone at the LAD ostium and limited commercially available Absorb BVS scaffold lengths. Ahead of a possible second scaffold distally (with minimal overlap), proximal optimisation was performed with a short 4.0 mm Hiryu (Terumo Medical, Tokyo, Japan) non-compliant monorail balloon. The jailed-wire was then removed having re-wired the diagonal branch through the LAD Absorb struts. In accordance with optimal Absorb BVS implantation technique, this was followed by post-dilation of the distal portion of the scaffold (beyond the side-branch) with a 3.5 mm Hiryu (Terumo Medical, Tokyo, Japan) non-compliant monorail balloon inflated to 20 atm.

**Fig. 2:**
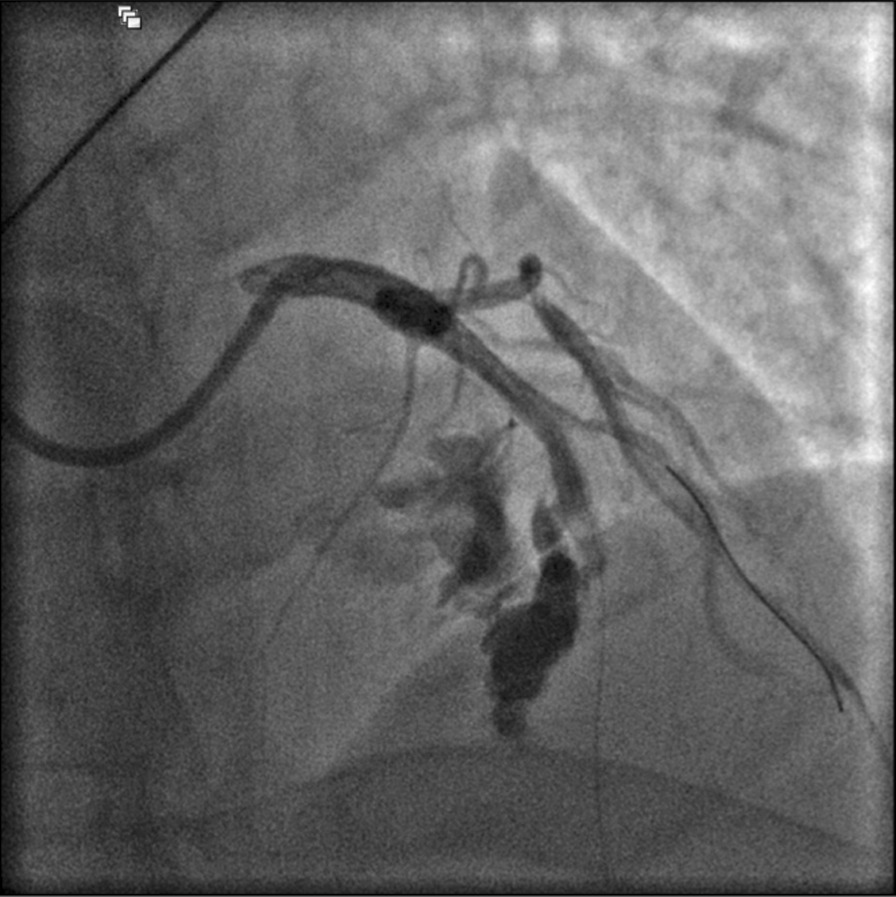
Large Ellis type III coronary perforation at distal edge of the BVS with no flow to distal LAD beyond the rupture. Prolonged balloon inflation immediately proximal to the site of perforation combined with fluid and vasopressor support achieved haemodynamic stability

**Fig. 3:**
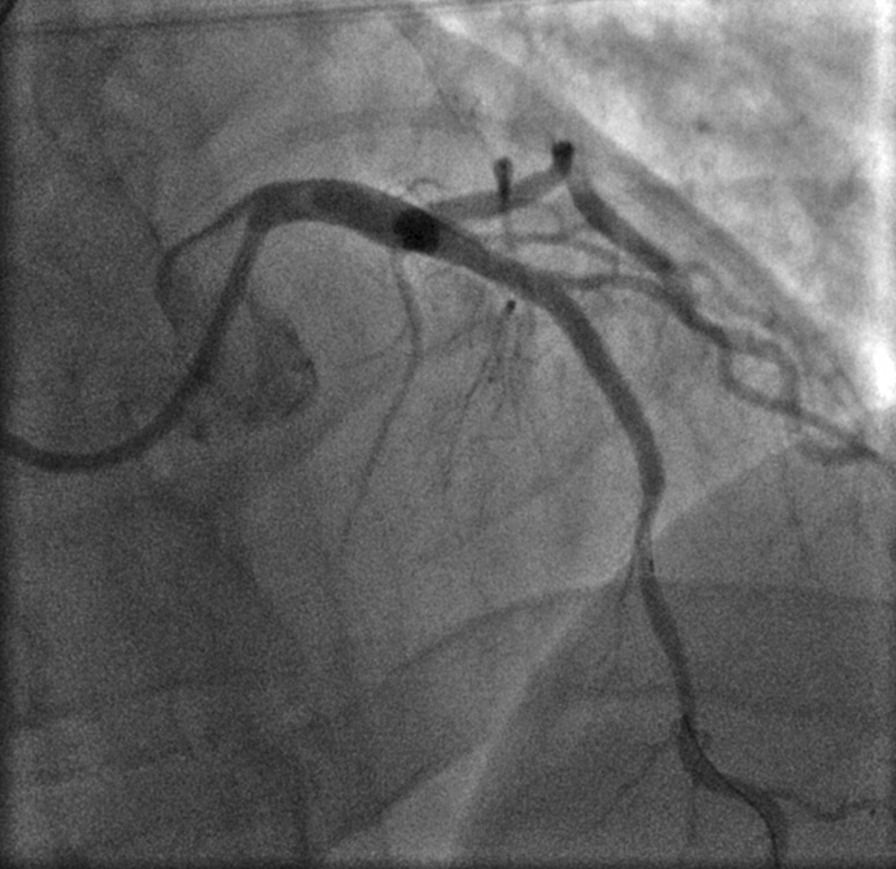
A 3.0 × 21 mm BeGraft covered stent deployed across the perforation distal to the diagonal side-branch at 14 atm, with approximately 10 mm of overlap between the Absorb BVS and the covered stent. Subsequent angiography demonstrated normal LAD flow and resolution of the perforation

Following post-dilation of the distal segment of the scaffold, widespread ST elevation and profound hypotension ensued. Angiography revealed a large, Ellis type III coronary perforation at the distal edge of the scaffold with no flow to the distal LAD beyond the rupture (Fig. 2), associated with a large pericardial effusion confirmed on bedside TTE. Prompt intravenous fluid and vasopressor support were administered and stability was initially re-established with a prolonged balloon inflation within the Absorb BVS immediately proximal to the site of perforation, permitting urgent insertion of a pericardial drain (Merit Medical, South Jordan, Utah, USA). 400 ml of blood was aspirated, with resolution of the hypotension, but despite this the chest pain and ST elevation persisted. As angiography during transient balloon deflation showed a large and persistent perforation, a 3.0 × 21 mm BeGraft covered stent (Bentley Innomed, Hechingen, Germany) was passed via the same right radial guide access and deployed across the perforation (distal to the diagonal side-branch) at 14 atm, with approximately 10 mm of overlap between the Absorb BVS and the covered stent. This lower profile device passed easily and smoothly through the Absorb BVS with no hold up. Covered stent implantation restored normal LAD flow angiographically and abolished the perforation (Fig. 3). The patient’s chest pain rapidly resolved and correspondingly the ST segments became isoelectric. Heparin reversal was not performed and cardio-pulmonary resuscitation was not required at any stage as despite initial profound hypotension, the patient remained conscious throughout the procedure. Haemodynamic stability was subsequently maintained and there was no re-accumulation of the pericardial effusion post-PCI. Sinus rhythm was maintained throughout with no arrhythmias noted during the event.

Notably, post-dilatation of the BeGraft covered stent was not performed despite the obvious size discrepancy between the 3.0 mm BeGraft covered stent within the 3.5 mm BVS, due to concerns with the risk of re-inflation re-opening the perforation, and subsequently a degree of malapposition was anticipated. No intracoronary imaging was performed during this procedure but a very good final angiographic result was achieved at the treatment site and without compromise of the diagonal side-branch (no side-branch or kissing balloon inflation was performed).

Repeat transthoracic echocardiography one day post-procedure demonstrated normal left ventricular systolic function and no residual pericardial effusion. His pericardial drain catheter was removed and he was transitioned from clopidogrel to prasugrel, and maintained on dual antiplatelet therapy to mitigate the risk of stent thrombosis due to assumed malposition at the site of BeGraft overlap within the larger Absorb BVS.

The patient was discharged 3 days later. In follow up, he remained asymptomatic and continued to demonstrate normal left ventricular systolic function on TTE. There was no evidence of inducible myocardial ischaemia at maximal workload on exercise stress echocardiography at 3 months.

**Fig. 4:**
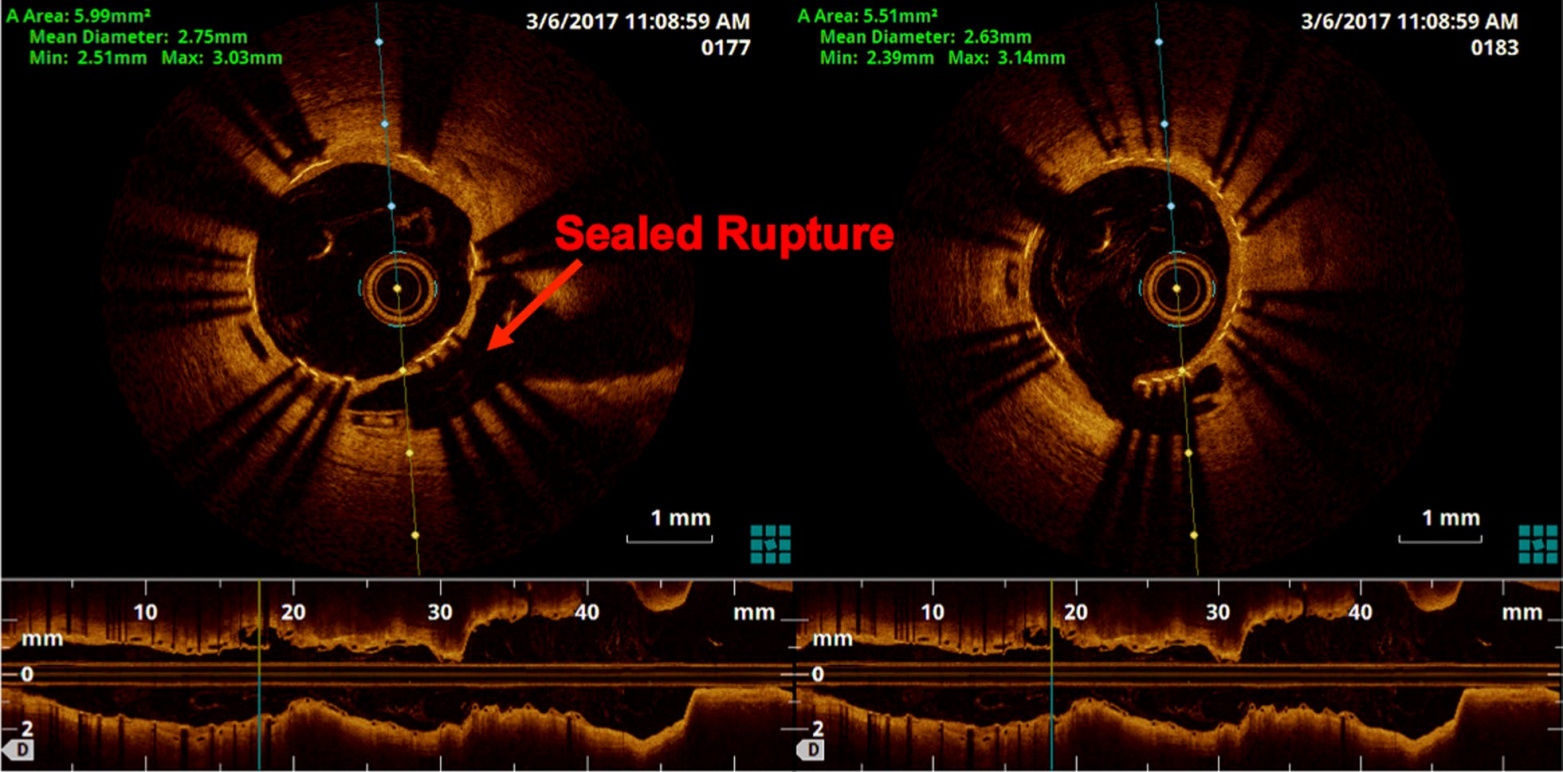
OCT demonstrating very short segment of malapposition of BeGraft in Absorb BVS at proximal overlap margin

**Fig. 5:**
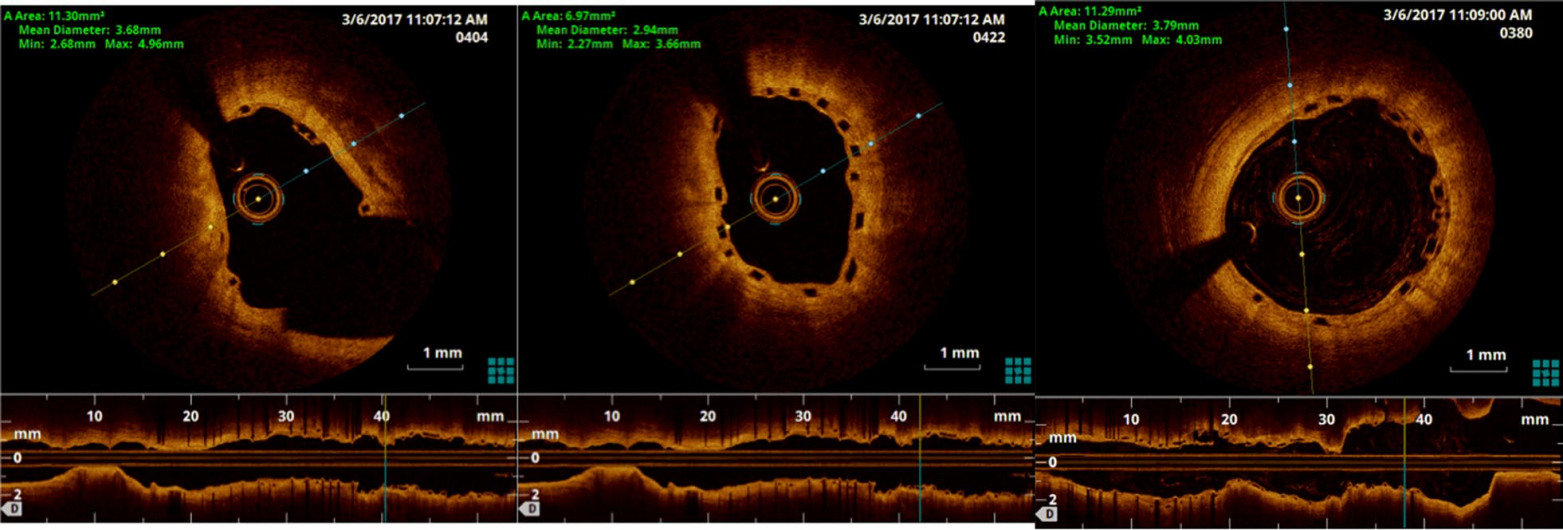
OCT demonstrating Absorb BVS well expanded proximal to BeGraft overlap

The patient returned for planned, repeat angiography with intracoronary imaging at 3 months. He had patent stents with TIMI III flow in the distal LAD and a disease free diagonal side-branch. The LCX and RCA were unchanged. Optical coherence tomography (OCT) with an Ilumien OPTIS PCI Optimisation (St Jude Medical, Saint Paul, Minnesota, USA) intravascular imaging system, showed good expansion and apposition of the Absorb BVS proximal to the BeGraft covered stent with incomplete strut coverage by neo-intimal tissue. There was a short segment of malapposition between the two devices at the proximal overlap margin adjacent to the area of sealed rupture and perforation (Fig. 4, Additional file 1: Fig. S5). There was otherwise good expansion and apposition of the BeGraft within the remaining distal overlap segment with the Absorb BVS but incomplete strut coverage (Fig. 5, Additional file 2: Fig. S7). There was evidence of neo-intimal hyperplasia and mild in-stent restenosis in the distal (non-overlap) portion of the Begraft covered stent. Given these findings, no further intervention was performed.

**Fig. 6:**
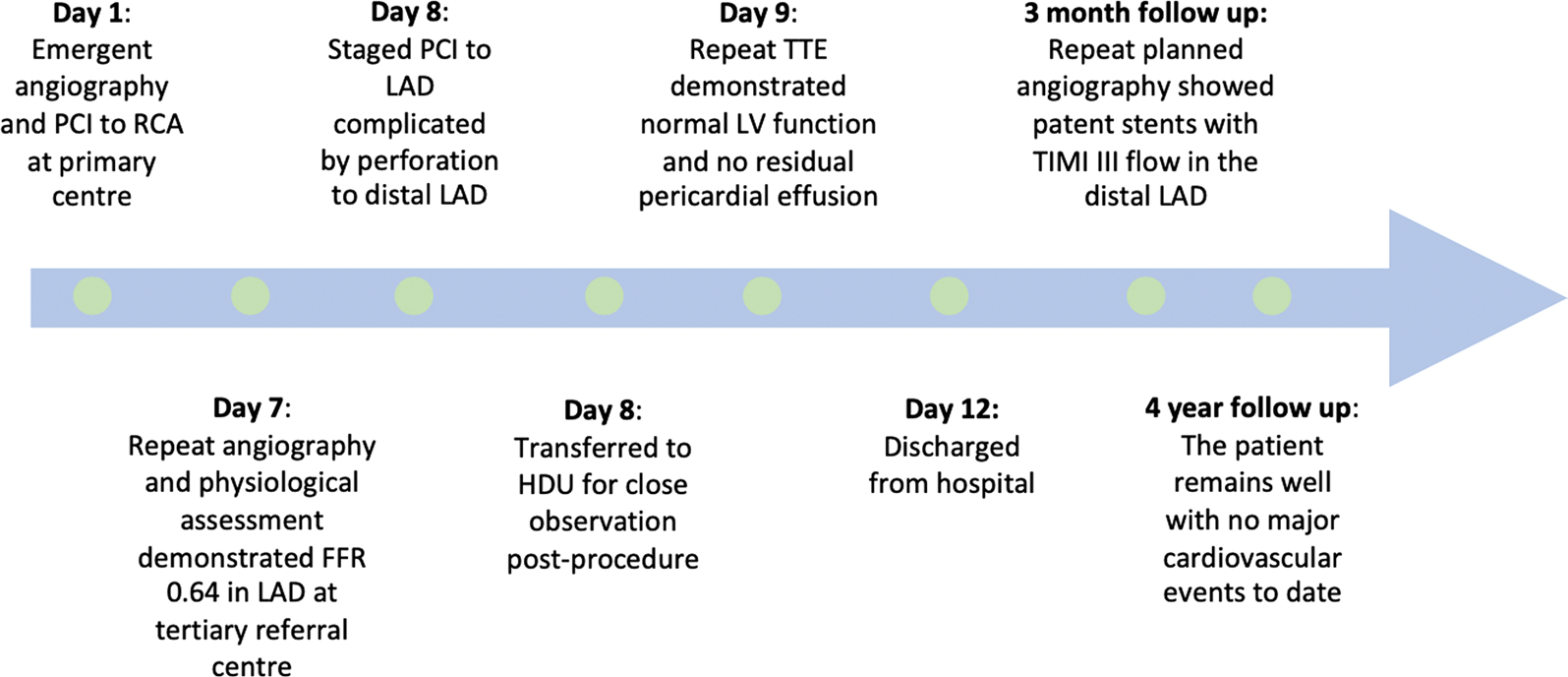
Timeline of events

The patient has remained well 4 years after PCI with no major cardiovascular events but still on DAPT (Aspirin and Clopidogrel) (Fig. 6).

## Discussion and conclusions

The underlying mechanism for coronary perforation in this case is likely to be multifactorial. The Absorb BVS implantation was performed using the ‘PSP’ method [4], which involves aggressive lesion preparation and adequate pre-dilatation to allow accurate sizing of the vessel, and post-dilatation at high inflation pressures to prevent both malapposition and under expansion of the Absorb BVS, and reduce the incidence of stent thrombosis [5]. Thicker Absorb BVS struts also exert a larger radial force. Additionally, intravascular imaging was not utilised to assess plaque composition and diameter stenosis after pre-dilation, which may have resulted in under recognition of coronary calcification and scaffold oversizing respectively; the clinical significance of coronary calcified nodules identified via intravascular imaging, and its association with coronary perforation has been demonstrated by Porto et al. in their clinical case report [6]. Subsequently, we postulate that the combination of high pressure balloon inflations and scaffold deployment, higher than recognised coronary calcification, and balloon and scaffold oversizing led to coronary artery perforation in this patient.

Despite the theoretical concerns with the ‘PSP’ method and the radial force of thicker scaffold struts, the incidence of coronary artery perforation complicating Absorb BVS implantation has not been defined in the literature, and only seven case reports discuss the management of this complication [7–12]. These cases utilised a variety of methods to achieve adequate sealing of the perforation, including prolonged balloon inflation, deployment of covered stents, insertion of pericardial drains, and heparin reversal.

In all three reported cases where covered stents were deployed [7, 8, 12], the polytetrafluoroethylene (PTFE) JOSTENT GRAFTMASTER covered stent (Abbott Vascular, Santa Clara, CA, USA) was used. A mesh-covered MGuard stent (MGuard, InspireMD, TelAviv, Israel) was trialled but failed in a fourth, separate case [7]. The use of a BeGraft covered stent in coronary artery perforation due to Absorb BVS, as in our case, has not been previously described in the literature.

Covered stents are effective treatment for coronary artery perforation when balloon inflation alone has not been able to seal the perforation and re-establish haemodynamic stability [13]. However, the higher profile of covered stents can be difficult to deliver, especially in small, torturous and calcified lesions [14]. The JOSTENT GRAFTMASTER, which consists of a single PTFE layer sandwiched between two stainless steel stents, has a crossing profile of between 1.63 mm and 1.73 mm. Newer generation covered stents, however, have been able to provide significantly better deliverability. In comparison to the older generation JOSTENT GRAFTMASTER, the BeGraft covered stent which we utilised has a single-layer design consisting of a PTFE membrane on a cobalt chromium steel platform, giving it a much slimmer crossing profile of 1.1 to 1.4 mm. Subsequently, we were able to deliver the BeGraft covered stent to the perforation site despite having had to cross thicker Absorb BVS struts. More recently, the PK Papyrus (Biotronik, Berlin, Germany) covered stent is another newer generation covered stent that received Food and Drug Administration (FDA) approval for clinical use in September 2018. It consists of a polyurethane membrane on a cobalt chromium steel platform with a similarly slimmer crossing profile of 1.18–1.55 mm.

Post-procedural intravascular imaging was used in two reported cases of perforation complicating Absorb BVS implantation for surveillance [7, 8]. In both cases, adequate stent apposition was confirmed. However, Freire observed, in his case, stent fracture of the BVS at both the time of implantation and again at 1 month, and the decision was made to deploy a DES over the BVS [7, 8]. In our case, when examined with OCT at 3 months, there was only a very limited area of malapposition between the Absorb BVS and the covered stent, and this did not need to be intervened upon.

This paper describes a case of coronary artery perforation complicating Absorb BVS implantation. In particular, we take away three key lessons. Firstly, this case highlights a potential hazard of the ‘PSP’ method and represents a salutary warning. Whilst meticulous implantation techniques for Absorb BVS including aggressive lesion preparation and very high pressure deployment and post-dilatation are associated with improved outcomes and reduced incidence of stent thrombosis, it can contribute to a higher risk of vessel perforation. Secondly, accurate sizing of the vessel is important in selecting the appropriate Absorb BVS. Intra-coronary imaging with OCT provides more accurate evaluation of the true target vessel reference dimensions and therefore in hindsight may have been beneficial in preventing this complication. Specifically, OCT may prevent the selection of an oversized scaffold and provide added diagnostic information regarding target lesion morphology, especially the extent and localization of calcification. This would both guide lesion preparation, but also potentially help predict the risk of perforation. Given these factors, even with newer generation thinner strut bioresorbable scaffold technologies, OCT guidance should probably be mandated for all implantations. Thirdly, and finally, newer generation covered stents with their single-layer design and slimmer crossing profiles have resulted in improved deliverability and procedural success, particularly when having to pass through thicker Absorb BVS struts. Whilst previous reported cases have utilised covered stents as part of the strategy to achieve adequate sealing of the coronary artery perforation, this case is the first in utilising the newer BeGraft covered stent, which was subsequently associated with excellent intra-coronary imaging appearance on follow up.

## Supplementary Information

**Additional file 1: Fig. S5**. OCT demonstrating very short segment of malapposition of BeGraft in Absorb BVS at proximal overlap margin (video).

**Additional file 2: Fig. S7**. OCT demonstrating Absorb BVS well expanded proximal to BeGraft overlap (video).

## Data Availability

Not applicable.

## Abbreviations

PCI: Percutaneous coronary intervention
LAD: Left anterior descending
LCx: Left circumflex
TTE: Transthoracic echocardiogram
NSTEMI: Non-ST elevation myocardial infarction
FFR: Fractional flow reserve
DES: Drug eluting stent
BVS: Bioabsorbable vascular scaffold
OCT: Optical coherence tomography
PTFE: Polytetrafluoroethylene
FDA: Food and Drug Administration
